# JMJD-5/KDM8 regulates H3K36me2 and is required for late steps of homologous recombination and genome integrity

**DOI:** 10.1371/journal.pgen.1006632

**Published:** 2017-02-16

**Authors:** Pier Giorgio Amendola, Nico Zaghet, João J. Ramalho, Jens Vilstrup Johansen, Mike Boxem, Anna Elisabetta Salcini

**Affiliations:** 1 Biotech Research and Innovation Centre (BRIC), University of Copenhagen, Copenhagen, Denmark; 2 Centre for Epigenetics, University of Copenhagen, Copenhagen, Denmark; 3 Developmental Biology, Department of Biology, Faculty of Science, Utrecht University, Padualaan 8, CH Utrecht, The Netherlands; MRC Laboratory of Molecular Biology, UNITED KINGDOM

## Abstract

The eukaryotic genome is organized in a three-dimensional structure called chromatin, constituted by DNA and associated proteins, the majority of which are histones. Post-translational modifications of histone proteins greatly influence chromatin structure and regulate many DNA-based biological processes. Methylation of lysine 36 of histone 3 (H3K36) is a post-translational modification functionally relevant during early steps of DNA damage repair. Here, we show that the JMJD-5 regulates H3K36 di-methylation and it is required at late stages of double strand break repair mediated by homologous recombination. Loss of *jmjd-5* results in hypersensitivity to ionizing radiation and in meiotic defects, and it is associated with aberrant retention of RAD-51 at sites of double strand breaks. Analyses of *jmjd-5* genetic interactions with genes required for resolving recombination intermediates (*rtel-1*) or promoting the resolution of RAD-51 double stranded DNA filaments (*rfs-1* and *helq-1*) suggest that *jmjd-5* prevents the formation of stalled postsynaptic recombination intermediates and favors RAD-51 removal. As these phenotypes are all recapitulated by a catalytically inactive *jmjd-5* mutant, we propose a novel role for H3K36me2 regulation during late steps of homologous recombination critical to preserve genome integrity.

## Author summary

DNA damage repair occurs in the context of chromatin and it is influenced by post-translational modifications of histone proteins. The level of methylation at lysine 36 of histone 3 (H3K36) plays an important role in early phases of DNA damage repair by recruiting early repair factors at the site breaks and regulating the formation of RAD-51 foci. Here, we suggest that the regulation of H3K36me2 levels by JMJD-5 is also relevant at later stages of DNA repair. By using *C*. *elegans* as model system, we show that loss of *jmjd-5*, resulting in increased levels of H3K36me2, impairs the resolution of recombination intermediates and the release of RAD-51, with consequences in the DNA repair process after ionizing radiation and in meiotic recombination. Thus, our work provides further insights into the mechanism regulating H3K36 methylation and its central role in repair of double strand breaks and in genome stability.

## Introduction

Cells have developed several pathways to promptly remove DNA lesions of different types in order to prevent dangerous outcomes such as cell death, DNA deletions and chromosomal rearrangements that can contribute to human diseases [1–3]. Double strand breaks (DSBs), involving both DNA strands, can be repaired by homologous recombination (HR), which uses the sister chromatid or the homologous chromosome as template to direct an error-free repair. Presynaptic steps of HR include the generation of ssDNA overhangs by DNA end resection and recruitment of the strand exchange protein RAD51 to the break site. This is followed by strand invasion of the ssDNA overhang into the DNA template (synapsis), formation of displacement loops (D-loops), and DNA synthesis [1]. Postsynaptic events of DSBs repair can proceed either with second DSB end capture and formation of Hollyday junctions (by the double Hollyday Junction pathway dHJ), or with the displacement of the invading strand and its ligation with the second end (by the SDSA pathway) [4]. Alternatively, DSBs can be repaired by the error-prone non-homologous end-joining pathway (NHEJ) [5]. NHEJ is based on protection of DNA ends mediated by the recruitment of Ku70/80 heterodimers and ligation of the ends by the DNA ligase 4/XLF/XRCC4 complex [6]. Though DSBs are generally deleterious, specific cell types self-inflict DSBs in particular conditions. This is the case in budding yeast during mating-type switching, in lymphocytes during V(D)J recombination or in germ cells during meiosis, in which DSB formation is a required step in the formation of crossovers between homologous chromosomes.

After DNA damage, cells activate a series of events, generally called DNA damage response, that includes the sensing of the damage, signaling and repair. In eukaryotes, the response to damage occurs in the context of chromatin in the cell nucleus, in which the DNA is wrapped around histone proteins [7]. Several studies highlight the relevance of chromatin components to properly execute DNA damage response and achieve successful repair. Over the last years, the role of histone variants, heterochromatic proteins and ATP-dependent chromatin remodeling factors in DNA damage response has been investigated in detail [8, 9]. Similarly, several histone modifications have been functionally linked to DNA damage repair [10–15]. In particular, the methylation state of histone H3 lysine 36 (lysines can be mono-, di- and tri-methylated) is emerging as a modification with key roles in the early phases of DNA repair. In human, tri-methylation of histone H3 lysine 36 (H3K36me3) is required for DNA damage sensing and DSB repair through HR, by promoting the recruitment of early components required for the formation of RAD51 foci [16–18]. Loss of the methyltransferase SETD2, responsible for H3K36me3 deposition, and overexpression of the H3K36me3-specific demethylase JMJD2A/KDM4A, inhibit DSB repair by HR. SETD2 and KDM4A members have also been implicated in DNA mismatch repair, in microsatellite instability, and in increased spontaneous mutation frequency, further indicating the relevance of this mark in genome stability [19, 20]. Likewise, di-methylation of H3K36 (H3K36me2) has been associated with early steps of the NHEJ repair pathway. After damage, H3K36 is rapidly di-methylated by the methyltransferase Metnase, which promotes NHEJ by recruiting early DNA repair components such as NBS1 and Ku70. Loss of Metnase and ectopic expression of JHDM1A, a KDM2 family member with specificity for H3K36me2, impair DSB repair by NHEJ [21, 22]. It is currently unknown if H3K36me2 also promotes HR-based repair. Furthermore, whether the modulation of H3K36 methylation is required after the initiation of DNA repair and for the progression of the repair process has not been investigated.

Considering the complexity and the dynamic nature of histone post-translational modifications, it is of crucial importance to systematically investigate the contribution of enzymes regulating histone post-translational modifications to DNA repair processes. *C*. *elegans* is an excellent model organism to uncover factors involved in induced-DNA damage repair, both in somatic and germ cells, by genetic screening approaches [23–25]. The mechanisms of DSBs formation and repair that occur physiologically in meiotic cells have been also studied in detail in the germline of *C*. *elegans* [26]. Importantly, DNA damage repair pathways are well conserved in the nematode and many of the components required in mammalian cells to ensure genome stability have also been identified in *C*. *elegans* as key molecules participating in responses to DNA damage [27].

In a screen to uncover potential roles of histone demethylases in the response to DSBs, we identified *jmjd-5* as a gene required for protecting germ cells from DSBs. JMJD-5 shares homology with the mammalian JMJD5/KDM8, a JmjC-containing protein essential for mouse embryonic development, cancer growth and mitotic division [28–32]. To date, the catalytic activity of JMJD5/KDM8 is debated, with reports supporting a role of this protein as a demethylase and others as a hydroxylase [29, 33–36].

Here we show that, in *C*. *elegans*, JMJD-5 regulates the level of H3K36me2 and it is required for DSB repair by the HR pathway. Animals carrying a deletion of *jmjd-5* are hypersensitive to IR and show prolonged retention of RAD-51 at DSBs, both after IR and during meiotic recombination. We found that *jmjd-5* genetically interacts with *rtel-1* and *helq-1*, which encode DNA helicases required for the resolution of postsynaptic recombination intermediates and the removal of RAD-51 from dsDNA-RAD-51 filaments, respectively [37, 38]. This suggests a postsynaptic role for JMJD-5 in regulating HR by promoting RAD-51 eviction and the progression of DNA repair. Importantly, the phenotypes observed in *jmjd-5* mutant animals are recapitulated in a catalytic inactive mutant, suggesting a novel and critical role for H3K36me2 regulation in late steps of DNA damage repair and in safeguarding genome integrity.

## Results

### *jmjd-5(tm3735)* animals are hypersensitive to IR

**Fig 1:**
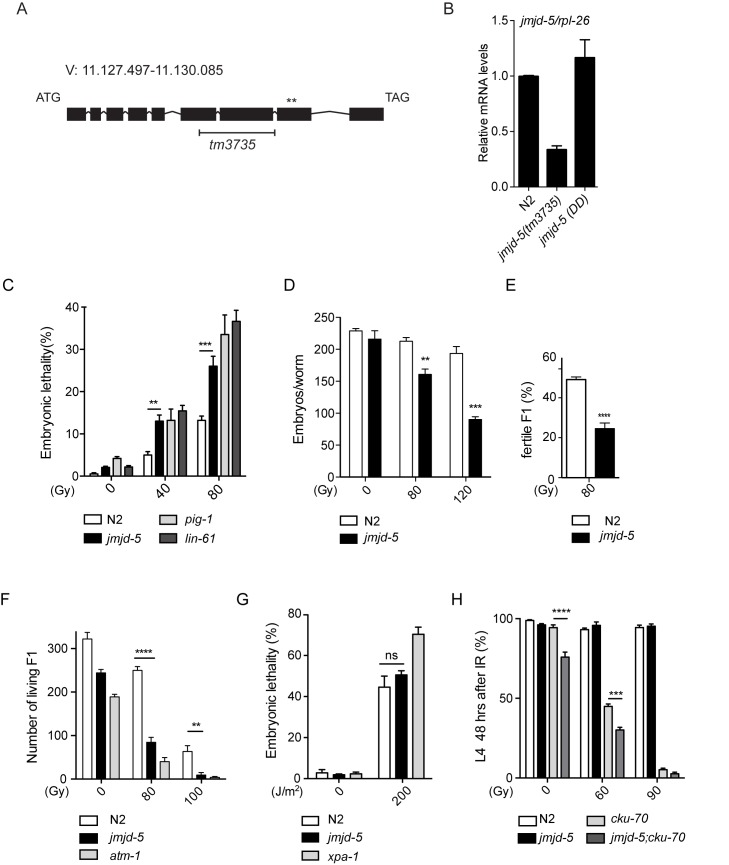
*jmjd-5* mutant animals are hypersensitive to IR. (A) Genomic structure of the *jmjd-5* gene. Dark boxes represent coding sequences and lines represent introns. The black H-shaped bar specifies the deletion in the *tm3735* allele and the asterisks indicate the position of the two amino acids (H484 and D486) located in the JmjC domain, mutagenized in *jmjd-5(DD)*. (B) Quantification of *jmjd-5* mRNA levels by qRT-PCR in the indicated strains. *rpl-26* is used as internal control. (C) Percent of embryonic lethality in the indicated strains observed after irradiation of young adult worms (24 h post L4) with 0, 40, or 80 Gy of IR. (D) Brood size in N2 and *jmjd-5(tm3735)* after IR treatment of young adults with 0, 80, or 120 Gy. Deposited embryos are counted from the time of irradiation. (E) Percentage of fertile animals in N2 and *jmjd-5(tm3735)* (evaluated by the presence of embryos in the uterus) among the living F1 progeny of irradiated mothers. (F) Number of living F1 animals in the indicated strains after irradiation of L1 larvae with 0, 80, or 100 Gy of IR. L1 were irradiated and the total number of living F1 offspring that passed the L1 stage is reported. (G) Percent of embryonic lethality in the indicated strains observed after treatment of young adult worms with 0 or 200 J/m^2^ of UV. (H) Percent of irradiated L1 of the indicated strains that developed to the L4 stage in 48 hours. In C-H, *pig-1(gm344)*, *lin-61(n3809)*, *atm-1(gk186)*, *cku-70(tm1524)* and *xpa-1(ok698)* are used as positive controls. In B-H, data are represented as means ± sem from at least 3 biological independent experiments. ****p< 0.0001, ***p<0.005, **p<0.01, n.s. = not significant, with two-tailed, unpaired t-test.

To identify potential histone demethylases involved in DSB repair, we performed a candidate screen of existing mutants of genes encoding JmjC-containing proteins. We identified *jmjd-5* as a gene required for normal response to ionizing radiation (IR), a source of DSBs. *jmjd-5* encodes for a 578 amino acid long protein and it is a member of the KDM8 family of proteins (Fig 1A and S1 Fig). The *jmjd-5(tm3735)* allele used in the screen carries a deletion of 621 bp plus an insertion of 2 bp in the *jmjd-5* gene (Fig 1A), which results in a significant decrease of the associated transcript level (Fig 1B). Analyses under standard growth conditions (20°C) revealed that *jmjd-5(tm3735)* animals are phenotypically wild-type, with only a slightly reduced brood size [average +/- sem: wild type (N2): 289+/-4, n = 25, *jmjd-5(tm3735)*: 231+/-19, n = 19] and very low level of embryonic lethality (Table 1).

**Table 1:** Embryonic lethality and male production in *jmjd-5* mutant animals. Embryonic lethality (Emb) and male production of the indicated genotypes were measured at 20°C and after growing the animals at 25°C for 5 generations.

Genotype	Emb (%)	Male (%)
N2 (20°C)	0.2 (n = 2119)	0.07 (n = 1485)
*jmjd-5(tm3735)* (20°C)	2.3 (n = 1057)	0.08 (n = 1303)
*jmjd-5(DD)* (20°C)	2.7 (n = 1475)	0.2 (n = 1475)
N2 (25°C)	1.8 (n = 4562)	0.2 (n = 3971)
*jmjd-5(tm3735)* (25°C)	11.8 (n = 2483)	3.2 (n = 1706)
*jmjd-5(DD)* (25°C)	11.7 (715)	2.3 (n = 1107)

However, when exposed to different doses of IR, adult *jmjd-5(tm3735)* animals showed increased embryonic lethality of their progeny (Fig 1C), increased chromosomal abnormalities in diakinesis (N2: 28.5%, n = 63, *jmjd-5*: 54.7%, n = 53, 80Gy) and reduced fertility, compared to N2 animals (Fig 1D). Moreover, the surviving *jmjd-5* F1 animals generated after the irradiation of adult animals, showed an increased rate of sterility (Fig 1E). Hypersensitivity to IR was also observed when we irradiated *jmjd-5(tm3735)* L1 larvae and measured their ability to produce viable progeny (Fig 1F). In contrast, the response to exposure to UV-C, known to generate lesions in only one DNA strand [39], was unaffected in *jmjd-5(tm3735)* animals, as the embryonic mortality rate was similar to that of N2 animals (Fig 1G). Overall, these results suggest that *jmjd-5* plays a protective role both in adult and larval germ cells towards double strand DNA damage. As the *C*. *elegans* germ cells favor HR-mediated repair, our data also support a role for *jmjd-5* in HR-mediated repair. To address whether *jmjd-5* also functions in NHEJ, an efficient but error-prone repair pathway activated mainly in somatic cells [40], we irradiated late embryos and tested their rate of growth. Irradiation of *jmjd-5* late embryos did not result in larval arrest, in contrast to animals lacking the NHEJ gene *cku-70*, suggesting that *jmjd-5* is not involved in NHEJ repair (Fig 1H). It should be noted, however, that the *cku-70; jmjd-5* double mutant has a low but significant larval arrest under normal conditions, suggesting that *jmjd-5* may have a protective role in somatic cells that is revealed when the NHEJ pathway is abrogated. In support of this possibility, *jmjd-5* was previously identified in a genome-wide RNA interference screen as required to protect genome stability in somatic cells [24].

In summary, loss of *jmjd-5* results in increased embryonic lethality and in decreased brood size after gamma irradiation, suggesting a role of *jmjd-5* in the HR-mediated repair process that protects germ cells from the consequences of DSBs generated by IR treatment.

### DNA damage checkpoints are activated normally in *jmjd-5* mutant animals

**Fig 2:**
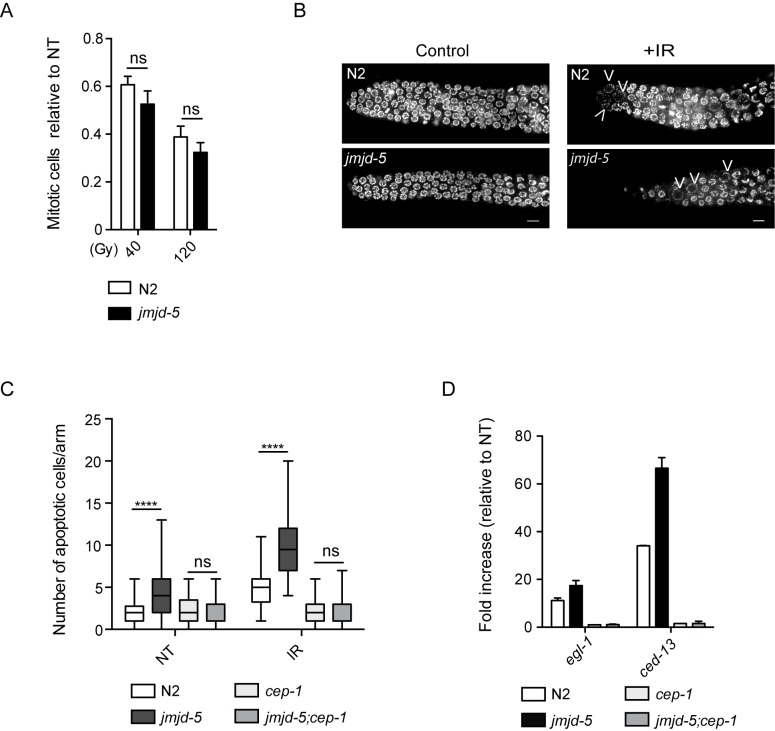
*jmjd-5* is not required for DNA damage checkpoints activation. (A) Number of mitotic cells in N2 and *jmjd-5(tm3735)* after irradiation of young adults with 40 and 120 Gy, counted 24 h after IR, relative to the non treated (NT) animals. (B) Representative images of germlines dissected from N2 and *jmjd-5* animals, 24 hours after irradiation with 0 (control) or 80 Gy (+IR), stained with DAPI. Arrowheads indicate arrested and enlarged nuclei. Scale bar: 5 μm. (C) Quantification of apoptotic cells in the indicated strains after staining with SYTO12, 24 hours after IR treatment (80Gy). NT, not treated. Between 40 and 78 animals were scored for genotype and condition. ****p< 0.0001, n.s. = not significant, with two-tailed, unpaired t-test. (D) Relative mRNA expression levels of the pre-apoptotic genes *egl-1* and *ced-13* in the indicated strains, 24 hours after IR treatment, measured by qPCR. Values are normalized to untreated samples and *act-1* is used as internal control. Graph is an average of two biological independent experiments ± sem. *cep-1(gk138)* is used as control.

After DNA damage, germ cells activate protective processes, including a block of mitotic cell division to allow DNA repair before replication, and the promotion of apoptosis to eliminate damaged cells. After IR, we observed a reduction in mitotic cell number in *jmjd-5(tm3735)* comparable to that detected in N2 animals (Fig 2A). This suggests that the checkpoint response to DNA damage in mitotic cells takes place normally in this mutant. Indeed, enlarged mitotic nuclei, generally regarded as a sign of mitotic arrest, are observed both in N2 and *jmjd-5(tm3735)* germlines after irradiation (Fig 2B). Likewise, apoptosis is activated in *jmjd-5(tm3735)* animals, as demonstrated by the presence of syto12-positive cells (Fig 2C) and increased transcript levels for the pro-apoptotic *cep-1*/p53-dependent genes *egl-1* and *ced-13* after IR (Fig 2D). Of note, *jmjd-5* mutant animals show increased apoptosis compared to wild-type animals not only after DNA damage induction by irradiation but also in untreated conditions (Fig 2C).

Consistently, removal of *cep-1*, required for eliminating damaged germ cells, in *jmjd-5(tm3735)* background is accompanied by a partial, but significant, restoration of fertility, by increased embryonic lethality and reduction of apoptosis (S2A–S2C Fig and Fig 2C and 2D).

These results indicate that checkpoint responses to IR are not defective in *jmjd-5* mutants, suggesting that the hypersensitivity to IR may be due to compromised DNA damage repair.

### *jmjd-5* is required for proper DNA damage repair

**Fig 3:**
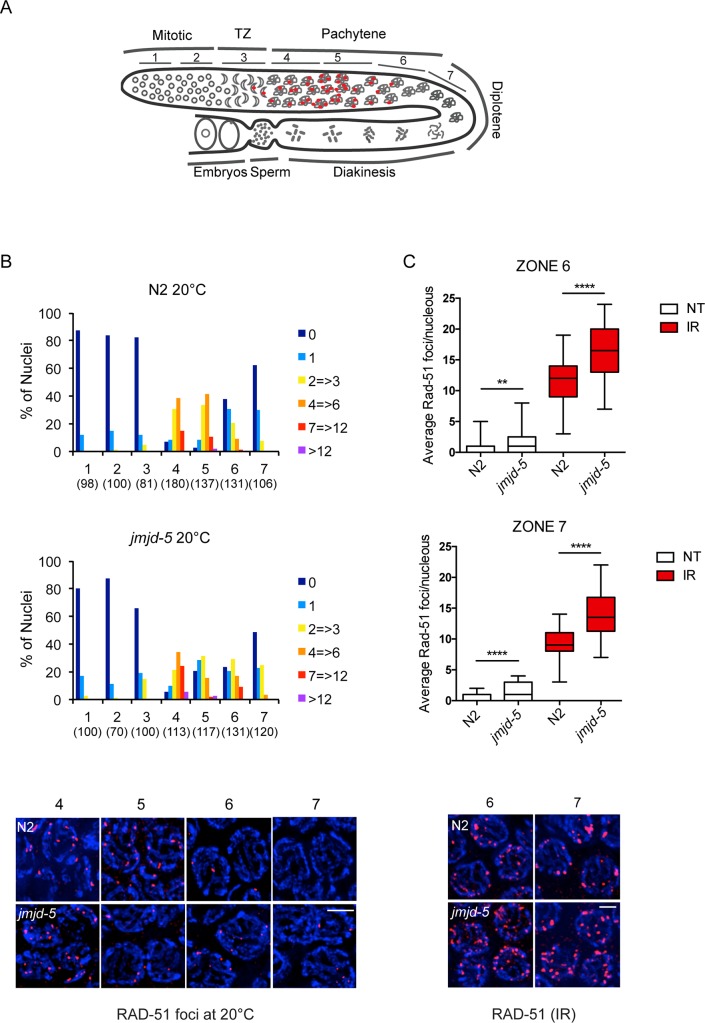
Aberrant RAD-51 staining in *jmjd-5* mutant animals. (A) Schematic representation of a *C*. *elegans* gonad arm, showing the position of the 7 zones scored for RAD-51 foci. TZ is the transition zone. Red dots represent RAD-51 foci distribution, according to literature. (B) Top. Histograms showing the quantification of RAD-51 foci in the extracted germlines of N2 and *jmjd-5(tm3735)* animals. Numbers in parenthesis indicate the number of nuclei analyzed. Bottom. Representative images of RAD-51 (red) and DAPI (blue) staining in germlines dissected from N2 and *jmjd-5(tm3735)* animals grown at 20°C. Nuclei from zones 4, 5, 6 and 7 are shown. (C) Top. Quantification of RAD-51 foci per nucleus in zones 6 and 7 of N2 and *jmjd-5(tm3735)* germlines, treated or not with IR (30Gy, 6 hours after the IR). At least 40 nuclei, from a minimum of 4 germlines for each genotype, were quantified for each zone and genotype. ****p< 0.0001, **p< 0.001, with two tailed unpaired t-test. Bottom. Representative images of RAD-51 (red) and DAPI (blue) staining in germlines dissected from N2 and *jmjd-5* animals, 6h after IR (30Gy). Nuclei from zones 6 and 7 are shown. In (B) and (C), each image shows a projection of multiple z-stacks (0.2 μm spacing) of the entire nuclei. 100X magnification, scale bar 2 μm.

DSB repair by HR is initiated by DNA-end resection followed by the recruitment of members of the recombinase family RecA/RAD-51 to the ssDNA. RAD-51 is a commonly used marker of DSBs whose recruitment at DNA breaks can be visualized as distinct bright foci by immunofluorescence microscopy. RAD-51 foci show a characteristic localization pattern in N2 germlines, as schematically shown in Fig 3A. Under physiological conditions, RAD-51 identifies DSBs required for meiotic recombination generated by *spo-11* [41]. RAD-51 foci arise in the transition zone (TZ, zone 3), where recombinant intermediates start to be coated by RAD-51, peak at early/mid pachytene (zone 4 and 5) and disappear in late pachytene (zone 6 and 7) [41, 42]. We first analyzed the pattern of RAD-51 staining in N2 and *jmjd-5(tm3735)* animals under normal conditions (Fig 3B). We found that the onset of RAD-51 staining was normal in *jmjd-5(tm3735)* animals and, despite a small decrease in the absolute number of RAD-51 foci in mid-pachytene (zone 5), the RAD-51 staining clearly arose in early and mid pachytene germ nuclei of *jmjd-5(tm3735)*. This suggests that the initial recruitment of RAD-51 on DSBs is unaffected in the mutant (Fig 3B). However, we observed that *jmjd-5(tm3735)* animals showed a small but consistent increase in the size of the region containing RAD-51 foci, with some foci persisting until the very end of pachytene, where they are normally absent in N2 (Fig 3B and 3C) (zone 6 *p*< 0.0001, zone 7, *p*<0.005, Student’s *t-test*, unpaired), suggesting that the *jmjd-5* mutant animals could encounter *spo-11*-independent unscheduled damage or have impaired repair of DSBs. To distinguish between these two possibilities, we analysed the staining of RAD-51 in *jmjd-5(tm3735);spo-11(me44)* genetic background. A dramatic decrease of RAD-51 staining was observed in both strains and no significant difference in the number of RAD-51 foci was observed in the double mutant compared to *spo-11* mutant (*p*>0.1, Student’s *t-test*, unpaired) (S3A Fig), indicating that the increase of RAD-51 foci observed in *jmjd-5* mutant is most likely related to defective DNA damage repair. Similarly, the increase of apoptotic cells observed in *jmjd-5* mutant animals (Fig 2C) was *spo-11*-dependent (S3B Fig). We repeated the RAD-51 analysis after IR and, as expected, both N2 and *jmjd-5(tm3735)* animals showed a large increase of RAD-51 staining and foci were observed in late pachytene (zone 6 and 7) also in N2. However, in *jmjd-5(tm3735)* animals, the average number of RAD-51 foci per nucleus in this region was significantly increased, compared to N2 (Fig 3C). We were unable to determine the exact number of RAD-51 foci in early/mid pachytene in this condition, due to the massive levels of RAD-51 staining.

Overall, these experiments show that *jmjd-*5 is likely not required at early steps of DNA repair, as RAD-51 is normally recruited to DSBs. Instead, *jmjd-5* mutant animals show a persistence of RAD-51 staining in late pachytene, rather supporting a role of *jmjd-5* in successfully completing DSB repair. Furthermore, the fact that aberrant persistence of *spo-11*-dependent RAD-51 foci is detected under normal conditions suggests a possible role for *jmjd-5* in HR occurring during meiosis.

### Role of *jmjd-5* in meiotic homologous recombination

**Fig 4:**
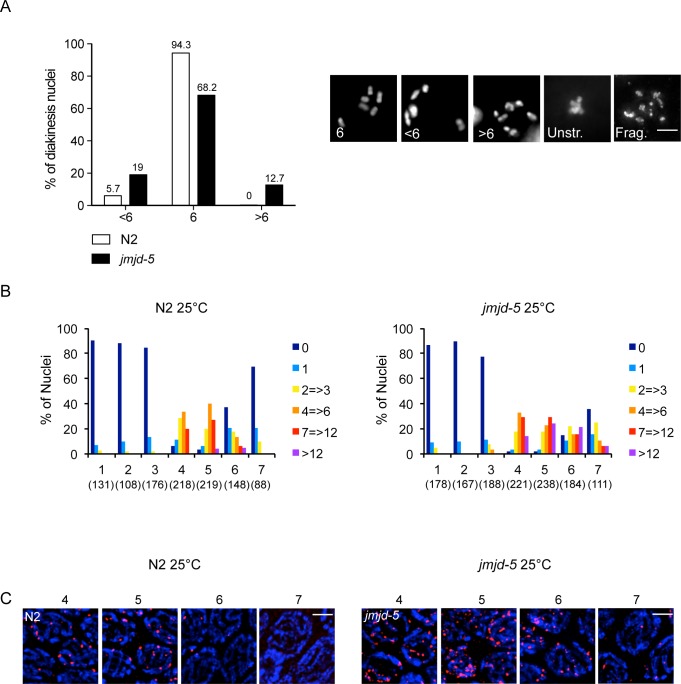
Role of *jmjd-5* in meiotic homologous recombination. (A) Left. Quantification of DAPI-stained bodies at diakinesis in N2 and *jmjd-5* mutants, grown at 25°C for five generations. Nuclei were divided in three classes based on the number of DAPI-stained bodies/nucleus, presented in the x-axis. The y-axis represents the percentage of nuclei in each class. Numbers of nuclei scored were 122 for N2 and 110 for *jmjd-5(tm3735)*. Right: representative images of DAPI-stained nuclei at diakinesis in N2 and *jmjd-5(tm3735)* mutants grown at 25°C for five generations. The number within each panel indicates the number of DAPI-stained bodies detected. Unstr: Unstructured chromosomes, Frag: Fragmented chromosomes. Scale bar 5 μm. (B) Histograms showing the quantification of RAD-51 foci in germlines extracted from N2 (left) and *jmjd-5(tm3735)* (right) animals grown at 25°C for five generations. Numbers in parenthesis indicate the number of nuclei analyzed. (C) Representative images of RAD-51 (red) and DAPI (blue) staining in dissected germlines of N2 (left) and *jmjd-5(tm3735)* (right) grown at 25°C for five generations. Nuclei from zones 4, 5, 6 and 7 are shown. In (A) and (C) each panel shows a projection of multiple z-stacks (0.2 μm spacing) of the entire nucleus. 100X magnification, scale bar 2 μm.

In addition to repair of DNA damage, HR is also required during meiosis. In this process, HR contributes to the formation of chiasmata, the structure that holds together the homologous chromosomes and ensures proper chromosome segregation in meiosis I. Failure to recombine results in chromosome misaggregation and aneuploid gametes, leading to embryonic lethality, as well as an increased number of males, generated by impaired X chromosome disjunction [43, 44]. We analyzed *jmjd-5(tm3735)* animals grown at 20°C and at 25°C, the latter being a temperature known to challenge the recombination process [45]. We did not observe signs of aberrant HR during meiosis at 20°C (Table 1), however, when animals were grown at 25°C for several generations (the data reported refer to the analysis of N2 and *jmjd-5(tm3735)* after five generations at 25°C), we noted decreased fertility (average +/- sem: N2: 185+/- 8, n = 12, *jmjd-5(tm3735)*: 78+/-13, n = 11) and a mild but significantly increased embryonic lethality and male production rates in the *jmjd-5(tm3735)* genetic background, compared to N2 (Table 1). In line with these latter phenotypes, we observed oocytes with aberrant numbers of paired chromosomes and, in some cases, with fragmented or compacted chromosomes (Fig 4A). The fact that these phenotypes appear after several generations at 25°C strongly indicates that they are not related to an intrinsic temperature sensitive property of the hypothetical mutated JMJD-5 protein. Instead, this observation suggests a protective role of *jmjd-5* in the germ cells at high temperature, which prompted us to visualize the pattern of RAD-51 staining at 25°C. In N2, the pattern of RAD-51 staining was qualitatively very similar to that observed at 20°C, with a clear reduction of RAD-51 foci in late pachytene (zone 6 and 7, compare Fig 3B and Fig 4B). We did observe a minor increase in the percentage of mid/late pachytene nuclei with higher numbers of RAD-51 foci (zone 5 and 6, *p*<0.005, Student’s *t-test*, unpaired), which could reflect the increased level of recombination occurring at this temperature, [45]. In contrast, in *jmjd-5(tm3735)* animals grown at 25°C the percentage of nuclei with high numbers of RAD-51 foci was increased compared to N2 cultivated at the same temperature (Fig 4B and 4C) (zones 4–7, *p*<0.0001, Student’s *t-test*, unpaired) and to *jmjd-5(tm3735)* grown at 20°C (compare Fig 3B and Fig 4B) (zone 4, *p*<0.005, zones 5–7, *p*<0.0001, Student’s *t-test*, unpaired). Furthermore, similarly to what we observed at 20°C, RAD-51 staining was also observed in a large percentage of nuclei in the late pachytene region of mutant germlines (Fig 4B and 4C). Persistent lesions derived by defects in meiotic DSB repair are reported to activate a meiotic checkpoint resulting in increased apoptosis [27, 46]. We therefore analyzed the rate of apoptosis in animals grown at 25°C and observed increased apoptosis in *jmjd-5(tm3735)* animals (S4 Fig) compared to wild type N2 in the same conditions, further confirming a role for *jmjd-5* in DSB repair.

The observation that RAD-51 foci persist to late pachytene in *jmjd-5* mutant animals at any temperature tested and after DNA damage, indicates a role for *jmjd-5* in steps occurring after RAD-51 loading and suggests that loss of *jmjd-5* results in HR intermediates loaded with RAD-51 that cannot be properly resolved, leading to increased apoptosis and chromosomal instability.

### *jmjd-5* facilitates postsynaptic RAD-51 removal

**Fig 5:**
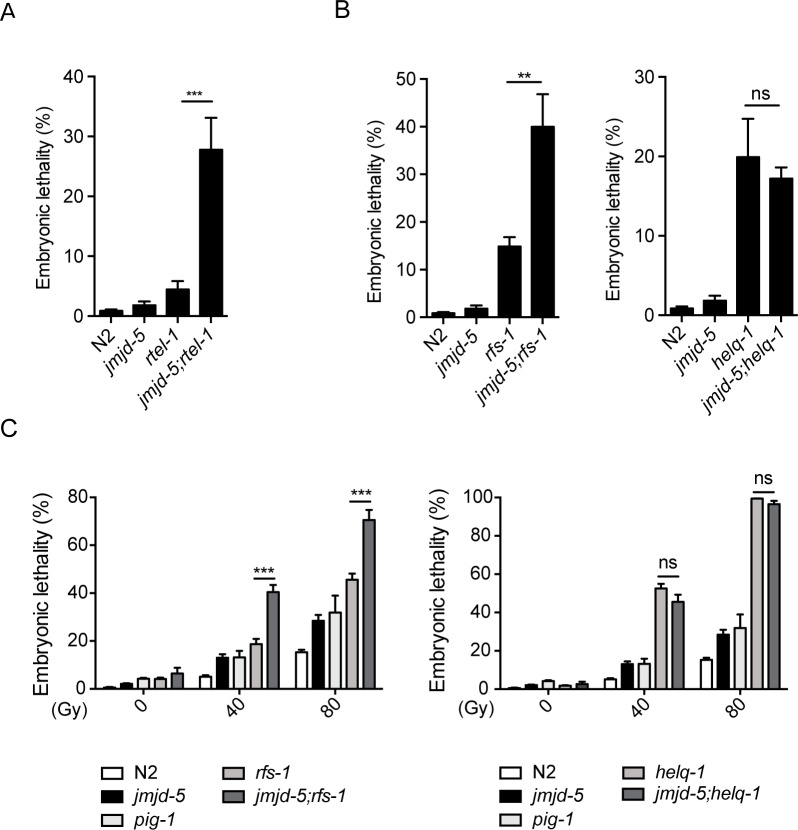
Genetic interaction analysis of *jmjd-5* with *rtel-1*, *helq-1* and *rfs-1*. (A) Percentage of embryonic lethality measured in the indicated strains, grown at 25°C for one generation. Embryos from adult animals grown at 20°C were plated at 25°C and their ability, once adult, to produce viable progeny was measured. (B) Percentage of embryonic lethality measured in the indicated strains, grown at 25°C for one generation as described in (A). (C) Percentage of embryonic lethality in the indicated strains after treatment of young adults worms with 0, 40 and 80 Gy. *pig-1(gm344)* is used as positive control. Data are represented as means ± sem from at least 3 biological independent experiments. ***p<0.005, **p<0.01, n.s. = not significant, with two-tailed, unpaired t-test.

To gain information regarding the site of JMJD-5 action, we used genetic approaches. JMJD-5 may act presynaptically, after RAD-51 recruitment on ssDNA, or postsynaptically, after D-loop formation. We postulated that if removal of *jmjd-5* results in increased stalled postsynaptic HR intermediates, the concomitant lost of *rtel-1*, a gene required for their resolution [37], should result in a worsening of the *jmjd-5* phenotypes. As *jmjd-5(tm3735)* shows embryonic lethality after some generations at 25°C, we measured the embryonic lethality of *jmjd-5;rtel-1* double mutants at this temperature. We found that, after only one generation at 25°C, embryonic viability was significantly affected, in comparison to single mutants (Fig 5A), indicating that loss of *jmjd-5* results in the formation of stalled HR intermediates that require *rtel-1* to be resolved. Considering that *rtel-1* disassembles RAD-51 double stranded DNA filaments (RAD-51-dsDNA) and has no activity on single stranded DNA-RAD-51 filaments (RAD-51-ssDNA) [37], our result also suggests that *jmjd-5* acts in a post-strand invasion step of HR.

The completion of HR events requires the eviction of RAD-51 from dsDNA, a process promoted, in *C*. *elegans*, by at least by two genes: *helq-1*, a helicase of the family of MUS308, and *rfs-1*, a paralog of RAD-51 [38]. *rfs-1* and *helq-1* act in two parallel and redundant pathways to remove RAD-51 from dsDNA filaments, as suggested by the previously reported evidence that the low level of embryonic lethality observed in single *helq-1* and *rfs-1* mutants dramatically increases in the *helq-1;rfs-1* double mutant [38]. The embryonic lethality observed in *helq-1;rfs-1* mutants is associated with stalled recombination intermediates, persistent RAD-51 foci in late pachytene, and defective chromosomal structures in diakinesis [38]. As these phenotypes are similar to the ones observed in *jmjd-5(tm3735)* animals raised at 25°C for several generations, we hypothesized that *jmjd-5* may act together with or in parallel to *helq-1* and *rfs-1* to promote RAD-51 removal. To test this hypothesis we generated *jmjd-5*;*helq-1* and *jmjd-5*;*rsf-1* double mutants and analyzed the level of embryonic lethality. We noted a significant increase of embryonic lethality in *jmjd-5;rfs-1* double mutants grown at 25°C for one generation, compared to the single mutants (Fig 5B). In contrast, *jmjd-5;helq-1* double mutants did not show an increase in embryonic lethality in this condition (Fig 5B), suggesting that *jmjd-5* acts in the same pathway as *helq-1* and in parallel to *rfs-1*. Furthermore, similar to *jmjd-5* mutants, animals carrying *rfs-1* and *helq-1* deletion alleles were previously found to be IR hypersensitive [47–49]. We therefore tested the *jmjd-5*;*helq-1* and *jmjd-5*;*rsf-1* double mutants for IR sensitivity. Again, loss of *rfs-1* acted synergistically with *jmjd-5*, as *jmjd-5;rfs-1* double mutants showed a remarkably increased sensitivity to IR, compared to single mutants. No additive effect was measured in *jmjd-5;helq-1* double mutant animals (Fig 5C). This further suggests that *jmjd-5* acts in the same pathway as *helq-1* and redundantly to *rfs-1* in protecting germ cells from IR.

Overall, these results suggest that *jmjd-5* acts postsynaptically, together with *helq-1* and in parallel to *rfs-1*, to facilitate RAD-51 removal from dsDNA, preventing stalled HR intermediate formation and therefore promoting successful completion of HR and DSBs repair.

### The catalytic activity of JMJD-5 is required in HR

**Fig 6:**
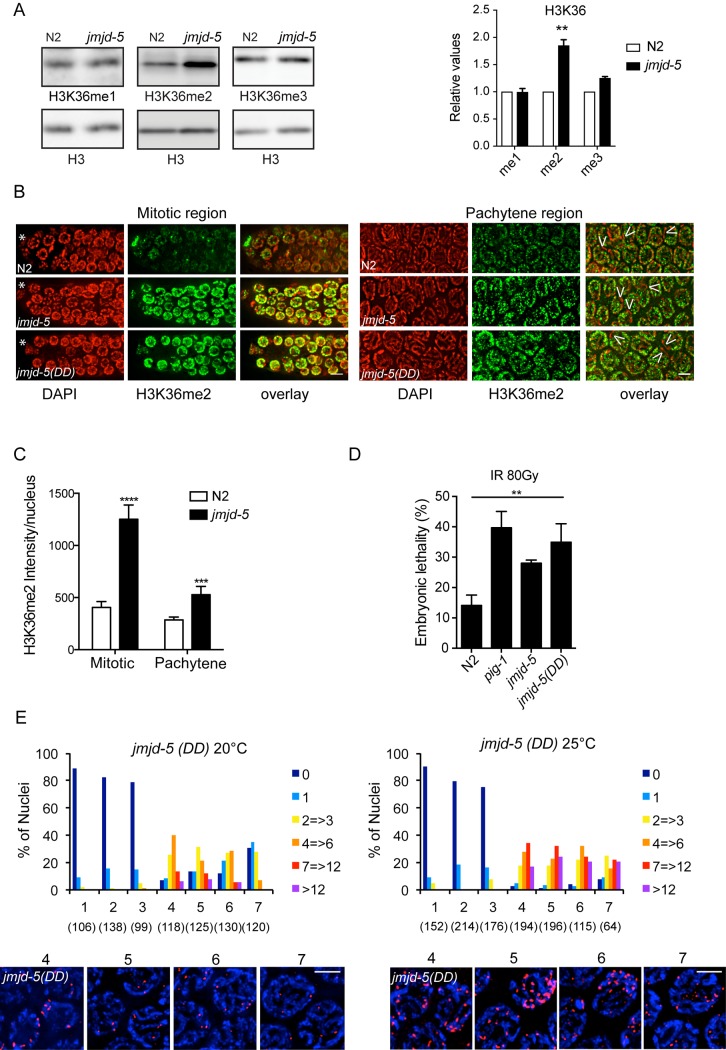
The catalytic activity of JMJD-5 is required for proper HR. (A) Left. Western blots of the indicated histone modifications using lysates from N2 and *jmjd-5(tm3735)* animals. H3 is used as loading control. Right. Quantification of H3K36 methylation levels, normalized to H3. Data are presented as a mean ± sem of three independent biological experiments. **p<0.01 with two tailed unpaired t-test. (B) Representative images of indicated germline regions of N2, *jmjd-5(tm3735)* and *jmjd-5(DD)* animals stained with DAPI (red) and H3K36me2 antibody (green). Overlay is on the right. Asterisks indicate distal tip cells, arrowheads indicate regions devoid of H3K36me2. (C) Quantification of average H3K36me2 intensity per nucleus in mitotic and pachytene regions of N2 and *jmjd-5* mutants. y-axis shows fluorescence intensity, expressed in arbitrary units. At least 10 gonads were quantified for each strain. Data are presented as mean ± sem. ****p< 0.0001, **p<0.01 with two tailed unpaired t-test. (D) Percent embryonic lethality in the indicated strains after treatment of young adults worms with 80 Gy. *pig-1(gm344)* is used as positive control. Data are represented as means ± sem of two independent biological experiments. **p<0.01 with two tailed unpaired t-test. (E) Top. Histograms showing the quantification of RAD-51 foci in germlines extracted from *jmjd-5(DD)* animals grown at 20°C (left) or at 25°C (right) for five generations. Numbers in parenthesis indicate the number of nuclei analyzed. Bottom. Representative images of RAD-51 (red) and DAPI (blue) staining in germlines of *jmjd-5(DD)* at 20°C (left) and 25°C (right). Nuclei from zones 4, 5, 6 and 7 are shown. In (B) and (E) each panel shows a projection of multiple z-stacks (0.2 μm spacing) of the entire nucleus. 100X magnification, scale bar 2 μm.

To investigate the function of *jmjd-5* in postsynaptic events, we analyzed its enzymatic activity. *jmjd-5* encodes a protein of 578 aa with a C-terminal JmjC domain, present in almost all known proteins with histone demethylase activity [50]. The specificity of the catalytic activity of the mammalian homologue, JMJD5/KDM8, is controversial, with several studies reporting specific H3K36me2 demethylase activity [29, 30, 34, 51], and others reporting a possible function as a protein hydroxylase [33, 36, 52]. We therefore tested if JMJD-5 is active towards H3K36me2 by measuring the level of this mark by western blot on lysates derived from adult *jmjd-5(tm3735)* animals. As shown in Fig 6A, loss of *jmjd-5* results in increased levels of H3K36me2, but not of H3K36me1/3. Other marks tested appear unchanged in the mutant (S5 Fig). As the *jmjd-5(tm3735)* phenotypes reported here are related to germ cell viability, we also analyzed the level of H3K36me2 in the germline using immunofluorescence (IF). As previously reported, in N2 germlines H3K36me2 is present in mitotic and meiotic germ cells with a dotted staining (Fig 6B), with a visible reduction in a region corresponding to the X chromosome [53]. Quantitative analysis revealed an increased level of H3K36me2 in mitotic and meiotic germ cells of *jmjd-5(tm3735)* animals (Fig 6B and 6C). Of note, the level of H3K36me2 in the somatic distal tip cell (Fig 6B) is not apparently increased, supporting a role for *jmjd-5* in regulating the H3K36me2 mark predominantly in germ cells. In addition, a region depleted of H3K36me2 staining that we assume to be, as in wild type N2 animals, the X chromosome, is still evident in the mutant, suggesting that JMJD-5 is most likely not contributing to the control of the H3K36me2 level on the X chromosome.

The JmjC domain contains a pocket that is required for catalytic activity and point mutations of residues located in this region are sufficient to abolish the enzymatic activity [54, 55]. Using CRISPR/Cas9-mediated genome engineering, we generated a mutant, termed *jmjd-5(DD)*, encoding a mutated protein in which two highly conserved amino acids of the pocket have been mutagenized (His484Asn and Asp486Ala) (Fig 1A). The point mutations generated did not affect the expression level of the gene, as judged by qPCR (Fig 1B). By immunofluorescence analysis, the level of H3K36me2 in *jmjd-5(DD)* appears increased compared to N2, further supporting a role of JMJD-5 in regulating the level of H3K36me2 (Fig 6B). To test whether the phenotypes observed in *jmjd-5(tm3735)* mutants are due solely to a lack of catalytic activity, we examined if *jmjd-5(DD)* animals show similar defects. We found that *jmjd-5(DD)* animals are hypersensitive to IR, as shown by increased embryonic lethality of F1 progeny of irradiated adults, compared to the control (Fig 6D). Furthermore, we observed a persistence of RAD-51 foci in late pachytene in the *jmjd-5(DD)* mutants compared to wild-type animals (Fig 6E, left) (zones 6–7, *p*<0.0001). Thus, the defects in RAD-51 staining observed in *jmjd-5(tm3735)* mutants are reproduced in *jmjd-5(DD)* animals. Strikingly, the RAD-51 defects were enhanced when *jmjd-5(DD)* animals were grown at 25°C for five generations (Fig 6E, right) (zone 4–7, p<0.0001, compared to wild-type in the same condition) and, consistently, *jmjd-5(DD)* animals cultivated at 25°C have increased level of males, of embryonic lethality (Table 1) and apoptosis (S4 Fig), indicating that the HR events occurring in meiotic cells are defective in the absence of the *jmjd-5* catalytic activity.

Overall these results suggest that JMJD-5 regulates H3K36me2 level and that the HR defects observed in *jmjd-5(tm3735)* mutant animals, both in physiological conditions and after irradiation, are strictly depending on the JmjC catalytic activity.

## Discussion

Here, we report a novel role for JMJD-5 in the process of HR, in response to DSBs occurring both under physiological conditions during meiosis, and after IR-induced DNA damage.

Our data suggest that *jmjd-5* is not required to mount DNA damage checkpoints, as mutant animals can properly elicit mitotic arrest and apoptosis when exposed to DNA damage. Similarly, in the absence of *jmjd-5*, RAD-51 is correctly loaded on DSBs, indicating that early steps of the DNA damage response (*e*.*g*., damage sensing, DNA resection and RAD-51 recruitment on ssDNA) are largely unaffected in the mutant strain. Our results point, instead, to a role for *jmjd-5* after RAD-51 recruitment that we characterize using genetic approaches. The observation that in *jmjd-5* mutant animals RAD-51 foci persist in late pachytene, together with the genetic interaction with *rtel-1*, which is required to resolve recombination intermediates, indicates that *jmjd-5* prevents the occurrence of stalled HR intermediates. Importantly, as *rtel-1* has no activity against RAD-51-ssDNA filaments, which resemble presynaptic HR substrates, but can unwind RAD-51-dsDNA filaments, this result also implies that *jmjd-5* acts in a postsynaptic step of HR. The genetic interaction of *jmjd-5* with *helq-1*, a gene required for the resolution of dsDNA RAD-51 filaments, further indicates that *jmjd-5* acts postsynatically, probably contributing to RAD-51 eviction and to the progression of the HR process, specifically acting in the *helq-1* pathway.

Our results show that the catalytic activity of JMJD-5 is critical during HR. Even though we cannot exclude the contribution of other unidentified targets, the upregulation of the levels of H3K36me2 in *jmjd-5* mutants indicates that the DNA damage-related functions of *jmjd-5* are directly correlated with the modulation of H3K36me2. It is possible that the *jmjd-5*-associated reduction of H3K36me2 facilitates RAD-51 eviction and the progression of DNA damage repair by favoring, directly or indirectly, the recruitment of HELQ-1. Alternatively, H3K36 methylation may compete with other modifications on H3K36 or on neighboring residues required for the completion of HR and DNA damage repair. In this context it is interesting to note that JMJD-5 carries at its N-terminal portion a GNAT (Gcn5-related N-acetyltransferase) domain, suggesting that JMJD-5 may be able to coordinate the levels of histone acetylation and methylation, favoring a more open chromatin environment that facilitates, for example, the helicase activity of HELQ-1 or DNA synthesis. As we failed to generate a mutant allele affecting specifically the GNAT domain, its activity, targets and implication in HR are at the moment unknown. We can, however, exclude that the increased H3K36me2 level observed after *jmjd-5* loss results in a DNA structure that is more sensitive to damage. Indeed, analysis of the level of RAD-51 foci one hour after irradiation, prior to DNA repair, resulted in a similar number of RAD-51 foci in N2 and *jmjd-5* mutant animals (S6A Fig). Similarly, loss of *jmjd-5* and the consequent increased level of H3K36me2 do not lead to remarkable changes in gene transcription, as suggested by the transcriptome analysis of *jmjd-5(3735)*, at 20°C and 25°C, obtained by RNA deep sequencing (S1 appendix), suggesting that the changes in H3K36me2 level do not largely impact transcription activity at global levels.

Despite we could not appreciate a global change of H3K36me2 level after irradiation (S6B Fig), a role of H3K36 methylation regulation in genome stability in *C*. *elegans* is also supported by studies on *jmjd-2*, an H3K9/K36me3 histone demethylase that has also been implicated in DNA damage response [55, 56]. Reduction of *jmjd-2* increases p53-dependent apoptosis and RAD-51 foci, in mid but not late pachytene [55], suggestive of a function of *jmjd-2* distinct from that of *jmjd-5*. The evidence that the mammalian homologue, JMJD2A/KDM4A, is also implicated in HR [18], suggests that the functions of H3K36 methylation regulation in DNA repair are evolutionary conserved and it is therefore tempting to speculate that the role of *jmjd-5* in late steps of HR that we identified in *C*. *elegans* is maintained in higher organisms. While direct evidence for such a role will require further studies in mammalian cell-culture systems, some indications that JMJD5/KDM8 is important for genome stability in mammals have been reported. JMJD5 was initially identified as a mutator gene in a screen using *Blm*^*m3*^ mice, carrying a hypomorphic mutation in the ReqQ-like gene 3 DNA helicase gene found in Bloom syndrome patients [31]. More recently, the catalytic activity of JMJD5 towards H3K36 has been suggested to ensure genome stability by preventing the formation of multipolar spindles in human cells [30].

It is noteworthy that some phenotypes of *jmjd-5* mutant animals appear after exposure to high temperature for several generations. Interestingly, as described for other chromatin factor mutants [57–59], *jmjd-5* mutant animals show progressive reduction of the brood size and could not be maintained at 25°C for many generations. Further analyses are required to understand the implication of defective DNA damage repair in this phenotype, nevertheless, these observations suggest that chromatin organization may be susceptible to temperature changes, which is also supported by studies in *Arabidopsis* and *Drosophila* [60–62]. They also suggest that the effects of aberrant histone modulation in germ cells can be transmitted to the progeny, leading to phenotypes that progress over generations.

In conclusion, our analysis provides novel information on JMJD-5 functions in HR and, by suggesting a previously uncharacterized role of H3K36 methylation in late steps of DNA damage repair, emphasizes the relevance of proper H3K36 methylation in the control of genome integrity.

## Materials and methods

### *C*.*elegans* culture and strains

*C*. *elegans* strains were grown at 20°C, unless stated otherwise, on NGM plates seeded with OP50 *E*. *coli* bacteria under standard conditions [63]. Strains used in this study were as follows: *jmjd-5(tm3735)*, NG4370: *zdIs5 I; pig-1(gm344) IV*, MT12833: *lin-61(n3809) I*, RB864: *xpa-1(ok698) I*, TJ1: *cep-1(gk138) I*, RB1279: *rfs-1(ok1372) III*, *helq-1(tm2134)*, FX1524: *cku-70(tm1524) III*, VC381: *atm-1(gk186) I*, GE24: *pha-1(e2123) III*, *rtel-1(tm1866)I*, ZR950: *jmjd-5(DD) V*, *AV157*:*spo-11(me44)/nT1 IV;V*. Double mutants were generated by standard crossing procedures. All experiments were conducted at 20°C, unless stated otherwise. The experimental protocols used in this work do not require an ethic statement.

### Genetic screen

*jmjd-5(tm3735)* was identified as hypersensitive to irradiation in a screen where mutants in JmjC-containing protein were tested. Briefly, synchronized young adult worms (24 hours post L4) of the strains indicated below were irradiated with 80Gy and the embryonic lethality of the F1 progeny deposited after 18–24 hours from the treatment was analysed. The following mutant alleles were tested: *rbr-2(tm3141)*, *rbr-2(ok2994)*, *jmjd-5(tm3735)*, *jmjd-4(tm965)*, *jhdm-1(tm2828)*, *jmjd-2(tm2966)*, *jmjd-1*.*2(tm3713)*, *jmjd-1*.*1(ba1083)*, *jmjd-3*.*1(gk387);jmjd-3*.*2(tm3121);jmjd-3*.*3 (tm3197)*. N2, *pig-1(gm344)* and *lin-61(n3809)* were used as controls in the screen. The screen was repeated three times.

### DNA damage sensitivity experiments

Synchronized young adult worms (24 hours post L4) of the indicated genotypes were treated with different doses of IR and UV. After treatments worms were allowed to recover for 18 hours. Embryonic lethality was assessed for the time period of 18–24 hours following treatment, by plating 3 worms per plate, in triplicate, per experiment. The number of dead embryos was assessed 24 hours later. The L1 survival assay was adapted from [64]. Briefly, L1 larvae of the indicated genotypes were irradiated and placed onto single plates (five worms per plate). The total number of living worms (post the L1 stage) present in the F1 generation was counted using a dissection microscope. Only plates in which all five irradiated P0 animals were alive were analyzed. For brood size measurement after different doses of IR, we counted the number of progeny (including dead embryos) produced after the irradiation time of synchronized young adult worms (24 hours post L4). Chromosome number and appearance in oocytes were analysed in DAPI stained animals after 18 hours of irradiation. At least three biological replicates were conducted for each experiment. In these experiments *pig-1(gm344)* [65], *lin-61(n3809)* [66], *atm-1(gk186)* [67], *cku-70(tm1524)* [68] and *xpa-1(ok698)* [69] were selected in base of their reported phenotypes and used, when indicated, as controls.

### Germ cell apoptosis assay

Adult animals (24 hours post L4) were irradiated and, after 24 hours were incubated in a 33 mM aqueous solution of SYTO12, for 4 hours at room temperature. After 45 minutes of recovery on OP50 plates, worms were then scored within 1 hour for the presence of fluorescent apoptotic cells. Only properly stained germlines were quantified. Acridine Orange staining (used in S3 Fig) was performed as previously described [70].

### Cell cycle arrest

Germlines of synchronized worms (24 hours post L4) were dissected after IR and stained with DAPI. Total number of mitotic cells was quantified in optically bisected gonads by fluorescence microscopy. At least 6 gonads were quantified per genotype and treatment dose.

### Fertility, male incidence and embryonic lethality analyses at 25°C

Embryos (P0) from animals of indicated genotypes were collected from adult animals grown at 20°C and plated and maintained at 25°C. Fertility, embryonic lethality and male production were estimated at the indicated generations.

### Immunofluorescence

Gonads of adult hermaphrodites (24 hours post L4) were dissected and stained as previously described [71]. Briefly, excised germlines were fixed for 10 minutes with 2% formaldehyde (Sigma Aldrich) in K2HPO4 (pH 7.2). Germlines were then freeze-cracked on dry ice and placed in cold methanol (Merck) for 5 minutes. Blocking was performed for 30 minutes in 1% BSA in PBST. Slides were incubated overnight at 4°C in a humid chamber with primary antibodies, washed 3 times for 10 minutes in PBST and incubated 2 hours with the secondary antibodies at room temperature. Slides were then washed 3 times for 10 minutes in PBST before being mounted on coverslips with Vectashield. In the second wash DAPI was added at a concentration of 100 ng/ml. Primary and secondary antibodies were diluted in blocking solution as follows: anti-RAD51 (Novus Biologicals; 29480002), 1:10000; anti-H3K36me2 (Active Motif; 61019) 1:200; donkey anti-mouse Alexa 488 (Life Technologies; A21202) 1:200, goat anti-rabbit Alexa 568 (Invitrogene; A11036) 1:200.

### Imaging and quantification

Immunofluorescence images were collected at 0.2 μm intervals using a Delta vision platform (GE Healthcare) with a IX-71 Olympus microscope Plan Apochromat, LED based 7 colour fluorescence illumination module (Lumencor) as light source, CoolSnap HQ2 camera (Photometrics), quadruple filter sets for DAPI, FITC, TRITC, and Cy5. Images were collected using a 100X oil objective (Olympus, 1.4NA) and three-dimensional data sets were subjected to deconvolution using softWoRx 6.5.2.Software Suite (Applied Precision) and then projected onto one dimension using ImageJ [72]. Exposure conditions were kept constant for each experiment. Entire germlines were reconstructed by stitching sequential images with ImageJ (ImageJ, National Institute of Health, Bethesda, MD) (Fiji).

Quantitative analysis of RAD-51 foci was performed as described in [46]. The number of RAD-51 foci/nucleus in the seven zones is divided in classes and reported in the x-axes of the histograms with a color code. The y-axis indicates the percentage of nuclei falling into each class. At least 5 germlines were scored for each condition. The total number of nuclei scored per zone is indicated in the histograms. Statistic analyses were performed using two tailed unpaired t test. Quantification of the average intensity of H3K36me2/nucleus was performed by measuring the intensity of selected regions in the germlines with ImageJ (Fiji) and by normalizing to the number of nuclei in that region. At least 10 gonads were quantified per genotype.

### Western blot

Western blots were performed as described in [73]. The following antibodies were used: anti-H3K36me1 (Abcam; ab9048) 1:1000, anti-H3K36me2 (Abcam; ab9049), 1:400; anti-H3K36me3 (Active Motif; 61102), 1:1000; anti-H3K27me3 (Millipore; 07–449), 1:2500; anti-H3K4me1 (Abcam; ab8895) 1:500, anti-H3K4me2 (Millipore; 07–030), 1:2000; anti-H3K4me3 (Abcam; ab8580) 1: 1000, anti-H3K9me3 (Abcam; ab8898) 1: 1000, anti-H3K27me2 (Abcam; ab24684) 1:2000, anti-H3 (Abcam; 1791), 1:30000. Quantification of intensity was performed using ImageJ software (ImageJ, National Institute of Health, Bethesda, MD).

### RNA sequencing and analysis

Young adult hermaphrodites cultured at the indicated temperatures and generations were flash-frozen in liquid nitrogen and stored at -80°C before RNA extraction. RNA was isolated from two independent cultures using TRIzol reagent (Life Technologies) and RNeasy Minikit (Qiagen). RNA amplification and sequencing were performed by the Beijing Genomics Institute (BGI). The samples were sequenced as 49 bp length single-end non-stranded reads on Illumina HiSeq2000 by BGI. The raw reads were mapped to the *C*. *elegans* ce10 genome assembly using the STAR alignment tool [http://bioinformatics.oxfordjournals.org/content/early/2012/10/25/bioinformatics.bts635] (v2.3.0) with default parameters (except: outFilterMismatchNoverLmax = 0.1, outFilterMatchNmin = 16). Uniquely mapped reads were assigned RefSeq genes by htseq-count [https://www.ncbi.nlm.nih.gov/pubmed/?term=25260700] (parameters: -a 30-s no -m intersection-nonempty). The RefSeq gene database was downloaded from UCSC (data stamped March 17 2013), ambiguously mapped genes removed and then filtered to keep only the longest transcript variant of a given gene. Statistical Wald tests (lfcThreshold = 0.5) were performed in R with the DESeq2 package (v1.12.4) [http://genomebiology.biomedcentral.com/articles/10.1186/s13059-014-0550-8] after first removing genes with no assigned reads. Differentially expressed genes were defined as having p-values corrected for multiple testing (Bonferroni) less than 0.1. Altogether slightly more conservative parameter setting than normal was chosen due to the limited number of biological replicates (two per group). Deregulated genes are listed in S1 appendix. Data are available in https://www.ncbi.nlm.nih.gov/geo/query/acc.cgi?acc=GSE93086.

### Real-time quantitative PCR (RT–qPCR)

Total RNA was isolated using TRIzol reagent (Life Technologies) and the cDNA was synthesized using TaqMan Reverse Transcription kit (Applied Biosystems). qPCR was performed as described in [74]. Reactions were performed in triplicate, in at least two independent experiments. Oligonucleotide sequences are available upon request. The housekeeping genes *rpl-26* and *act-1* were used as internal controls.

### Generation of *jmjd-5(DD)* by CRISPR/Cas9

The *jmjd-5(DD)* strain was generated by microinjection of young adult *pha-1(e2123)* worms using the co-CRISPR approach described in [75]. The injection mix contained plasmid pJW1285 that drives expression of Cas9 and *pha-1(e2123)* sgRNA (60ng/μL, Addgene#61252), the *pha-1(e2123)* PAGE-purified 80mer single-stranded oligodeoxynucleotide (ssODN) HR template (50ng/μL; IDT), two plasmids expressing sgRNAs targeting the *jmjd-5* locus (50ng/μL each), and an 100mer ssODN HR template to introduce the desired mutations in *jmjd-5* (100ng/μL; IDT). To generate sgRNA plasmids targeting the *jmjd-5* locus annealed oligo pairs were ligated into BbsI-digested pJJR50 (Addgene#75026). To identify worms with edited *jmjd-5* alleles surviving F1 progeny of injected animals was singled and allowed to lay eggs followed by lysis with 120ug/mL Proteinase K (Macherey-Nagel). Lysates were used as input in PCR reactions using the DD F/R primer pair and results were confirmed by Sanger sequencing of the DD seq F/DD R amplicon. Progeny of positive integrants was backcrossed once with N2 males to remove the remaining *pha-1(e2123)* allele, which was confirmed by Sanger sequencing of the *pha-1* F1/R1 amplicon. Sequences of the oligonucleotides used for the generation of *jmjd-5(DD)* are reported in S1 Table.

## Supporting information

S1 Fig JMJD-5 ortholog tree. Recursive blastp alignments against the 'non-redundant protein sequences (nr)' database were performed based on JMJD-5 *C*.*elegans* full length protein sequence NP_505831. The alignment search space was limited to *Caenorhabditis*, *Homo sapiens*, *Mus musculus*, *Gallus gallus*, *Canis lupus familiaris*, *Danio rerio*, *Drosophila melanogaster and California sea hare* (*Aplysia californica*). The best alignment hit of each species was noted, and if two sequences was each others best hit, they were regarded as putative orthologs. The protein sequences of identified orthologs were retrieved and a multiple alignment performed using M-Coffee (http://tcoffee.crg.cat, default settings). The alignment was imported into R and with the 'phangorn' package a tree with non-parametric bootstrap confidence values were calculated and plotted (LG substitution matrix, neighbor-joining).
(TIF)

S2 Fig Fertility, embryonic lethality and apoptosis in irradiated *jmjd-5* mutant depend on *cep-1*. (A) Brood size of the indicated strains after irradiation of young adults with 120 Gy. Embryos are counted from the time of irradiation. (B) Embryonic lethality in the indicated strains with or without irradiation (80Gy). (C) Representative images of SYTO 12 staining of gonads extracted from irradiated animals of the indicated strains.
(TIF)

S3 Fig *spo-11*-dependent RAD-51 foci and apoptosis. (A) Histograms showing the quantification of RAD-51 foci in the extracted germlines of *spo-11(me44)* and *jmjd-5(tm3735);spo-11(me44)* animals. Numbers in parenthesis indicate the number of nuclei analyzed. (B) Quantification of apoptotic germ cells in the indicated strains measured using Acridine Orange staining. At least 43 animals were scored for each genotype. ****p< 0.0001, n.s. = non significant with two tailed unpaired t-test.
(TIF)

S4 Fig Apoptosis in *jmjd-5* mutant animals at 25°C. Quantification of apoptotic germ cells in the indicated strains grown at 25°C for five generations, using SYTO-12 staining. The average amount of SYTO12-labeled apoptotic corpses per gonad arm is normalized to the average number of meiotic germ cells in each of the indicated genotypes. At least 20 animals were scored for each genotype. ****p< 0.0001, ***p< 0.005 with two tailed unpaired t-test.
(TIF)

S5 Fig Histone modifications in *jmjd-5(tm3735)* and *jmjd-5(DD)*. (A) Representative Western blots of the indicated histone modifications using lysates from N2 and *jmjd-5(tm3735)* animals. (B) Western blots of the indicated histone modifications using lysates from N2 and *jmjd-5(DD)* animals. H3 is used as loading control. Quantified levels of the histone modifications in *jmjd-5* mutants relative to N2, are indicated. Quantification was performed using ImageJ. ± indicates SD, calculated from at least two independent experiments.
(TIF)

S6 Fig DNA lesions soon after IR in N2 and *jmjd-5(tm3735)* animals and H3K36me2 levels after IR. (A) Left: Representative images of RAD-51 (red) and DAPI (blue) staining in dissected germlines of N2 and *jmjd-5(tm3735)* 1 hour after IR (30 Gy). Mitotic and pachytene regions are shown. Each panel shows a projection of multiple z-stacks (0.2 μm spacing) of the entire nuclei. 100X magnification, scale bar 5 μm. Right: Quantification of RAD-51 foci per nucleus in N2 and *jmjd-5(tm3735)* 1 hour after IR (30Gy) in mitotic and pachytene regions. At least 4 germlines were analyzed. (B) Western blots showing the global level of H3K36me2 in N2 treated (IR) or not (NT) with 80Gy. Animals were collected after 1 and 24 hours of the treatment. H3 is used as loading control. Samples from two independent experiments are presented.
(TIF)

S1 Table Oligonucleotides used for the generation of the *jmjd-5(DD)* mutant. (TIF)

S1 Appendix Deregulated genes in *jmjd-5(tm3735)*. List of genes differentially expressed in *jmjd-(tm3735)*, compared to wild type. RNA from young adult hermaphrodites cultured at the indicated temperatures and generations was extracted and processed as described in Material and Methods. Differentially expressed (DE) genes were defined as having *p*-values corrected for multiple testing (Bonferroni) less than 0.1. DE_G0 (Differentially expressed genes at 20°C), DE_G1 (Differentially expressed genes after 1 generation at 25°C) and DE_G6 (Differentially expressed genes after 6 generations at 25°C).
(XLS)
