# Supplementary figures and images for: JMJD-5/KDM8 regulates H3K36me2 and is required for late steps of homologous recombination and genome integrity
Source: PLoS Genet. 2017 Feb 16;13(2):e1006632. doi: 10.1371/journal.pgen.1006632 (PMC5336306; doi:10.1371/journal.pgen.1006632)

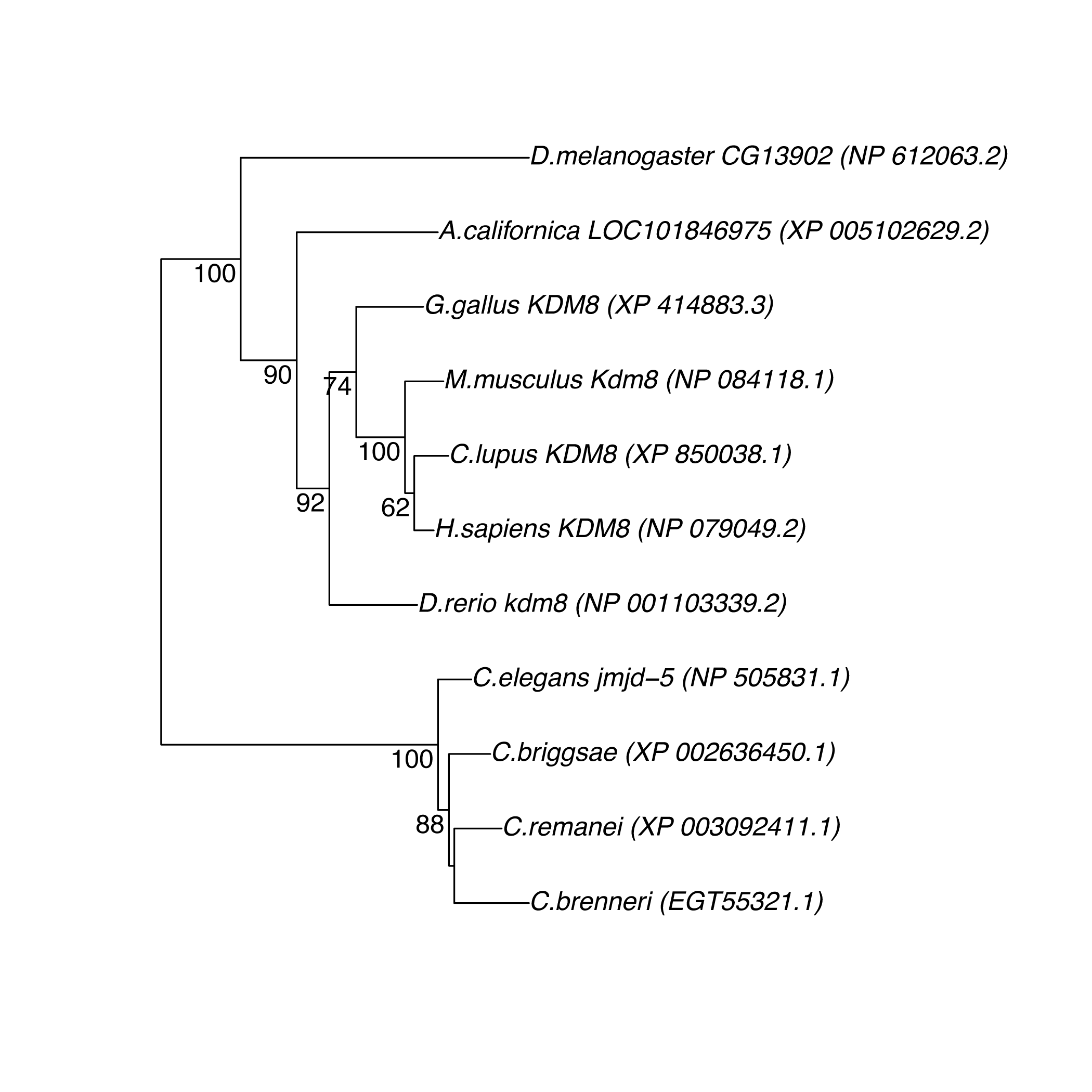

Supplement: S1 Fig — Recursive blastp alignments against the 'non-redundant protein sequences (nr)' database were performed based on JMJD-5 C.elegans full length protein sequence NP_505831. The alignment search space was limited to Caenorhabditis, Homo sapiens, Mus musculus, Gallus gallus, Canis lupus familiaris, Danio rerio, Drosophila melanogaster and California sea hare (Aplysia californica). The best alignment hit of each species was noted, and if two sequences was each others best hit, they were regarded as putative orthologs. The protein sequences of identified orthologs were retrieved and a multiple alignment performed using M-Coffee (http://tcoffee.crg.cat, default settings). The alignment was imported into R and with the 'phangorn' package a tree with non-parametric bootstrap confidence values were calculated and plotted (LG substitution matrix, neighbor-joining). (TIF) [file pgen.1006632.s001.tif]

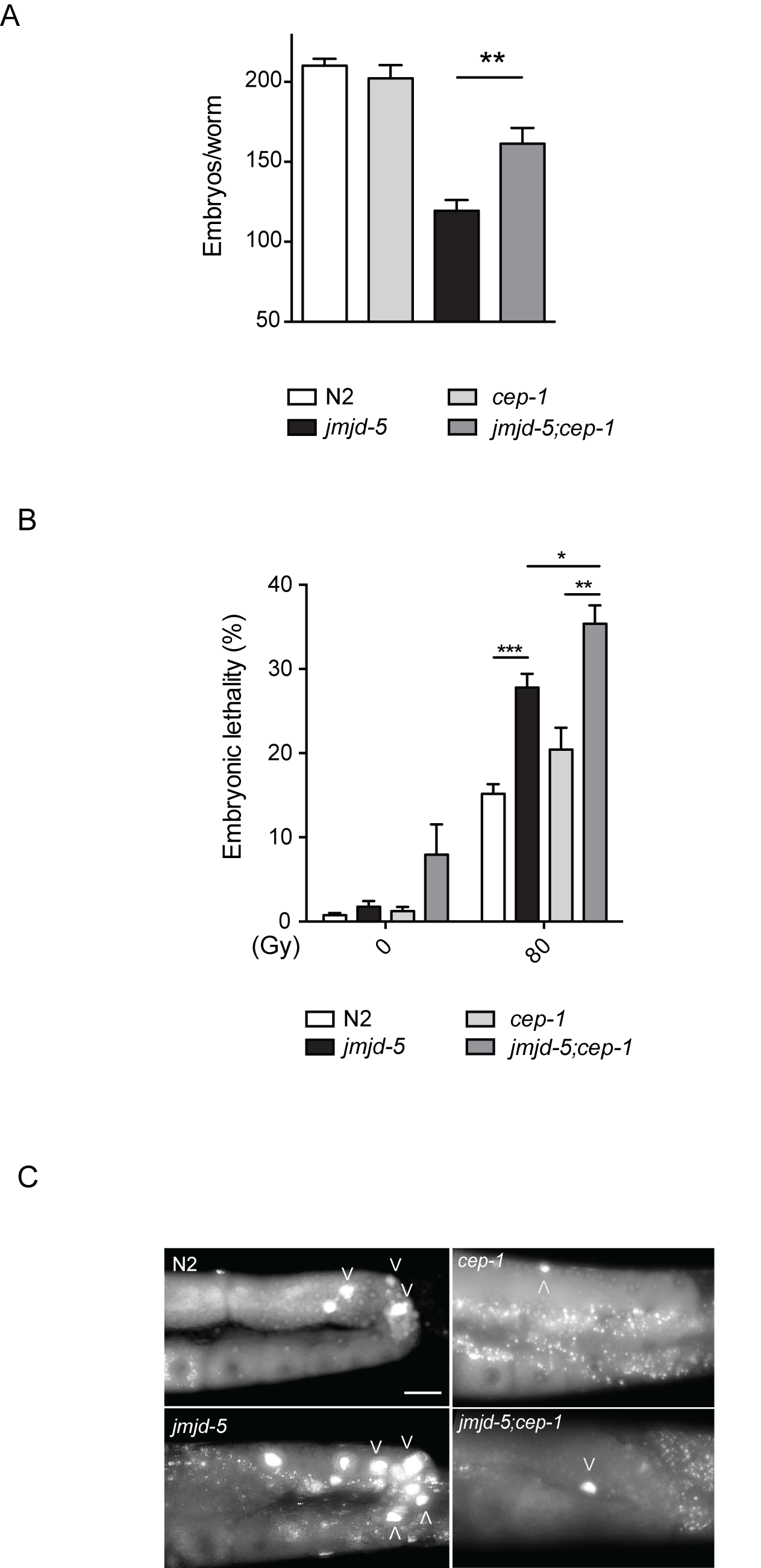

Supplement: S2 Fig — (A) Brood size of the indicated strains after irradiation of young adults with 120 Gy. Embryos are counted from the time of irradiation. (B) Embryonic lethality in the indicated strains with or without irradiation (80Gy). (C) Representative images of SYTO 12 staining of gonads extracted from irradiated animals of the indicated strains. (TIF) [file pgen.1006632.s002.tif]

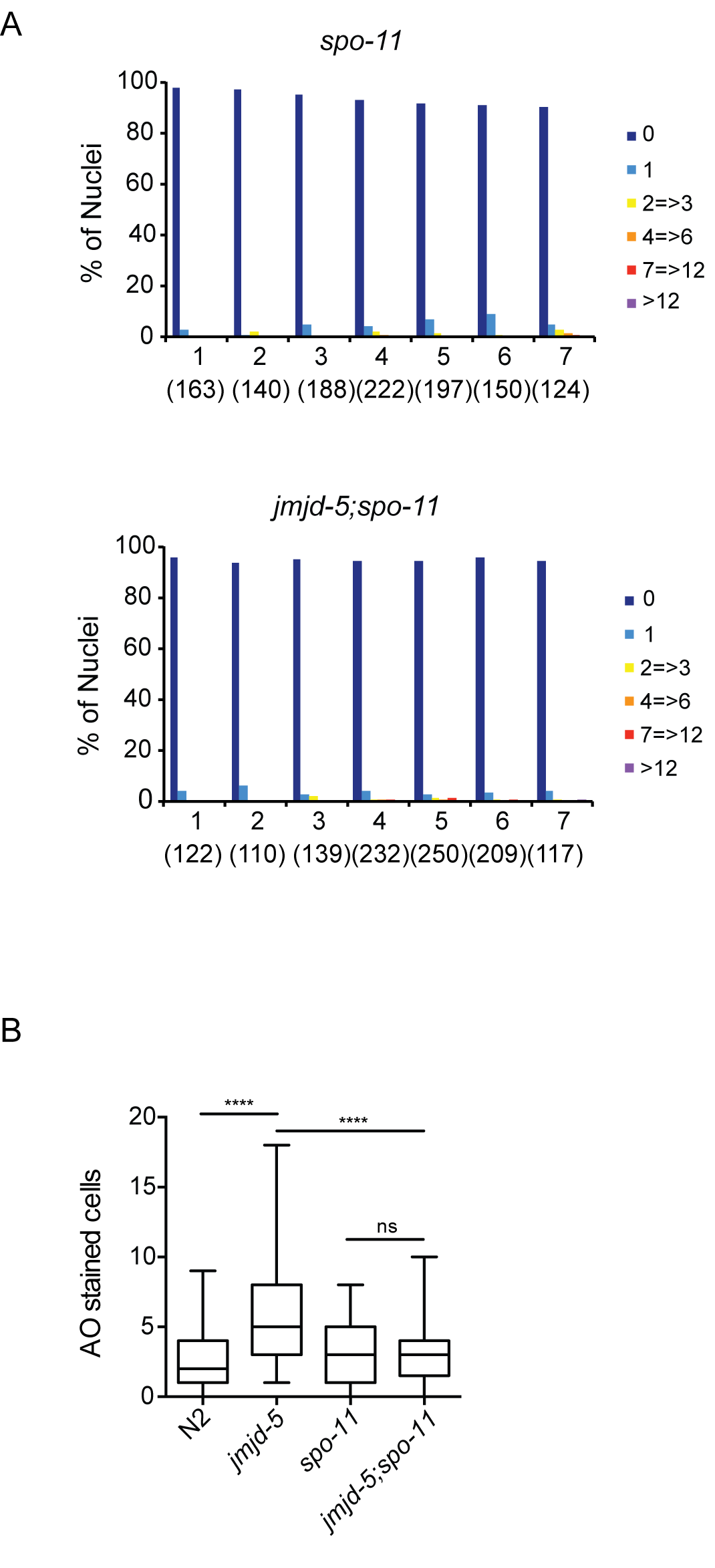

Supplement: S3 Fig — (A) Histograms showing the quantification of RAD-51 foci in the extracted germlines of spo-11(me44) and jmjd-5(tm3735);spo-11(me44) animals. Numbers in parenthesis indicate the number of nuclei analyzed. (B) Quantification of apoptotic germ cells in the indicated strains measured using Acridine Orange staining. At least 43 animals were scored for each genotype. ****p< 0.0001, n.s. = non significant with two tailed unpaired t-test. (TIF) [file pgen.1006632.s003.tif]

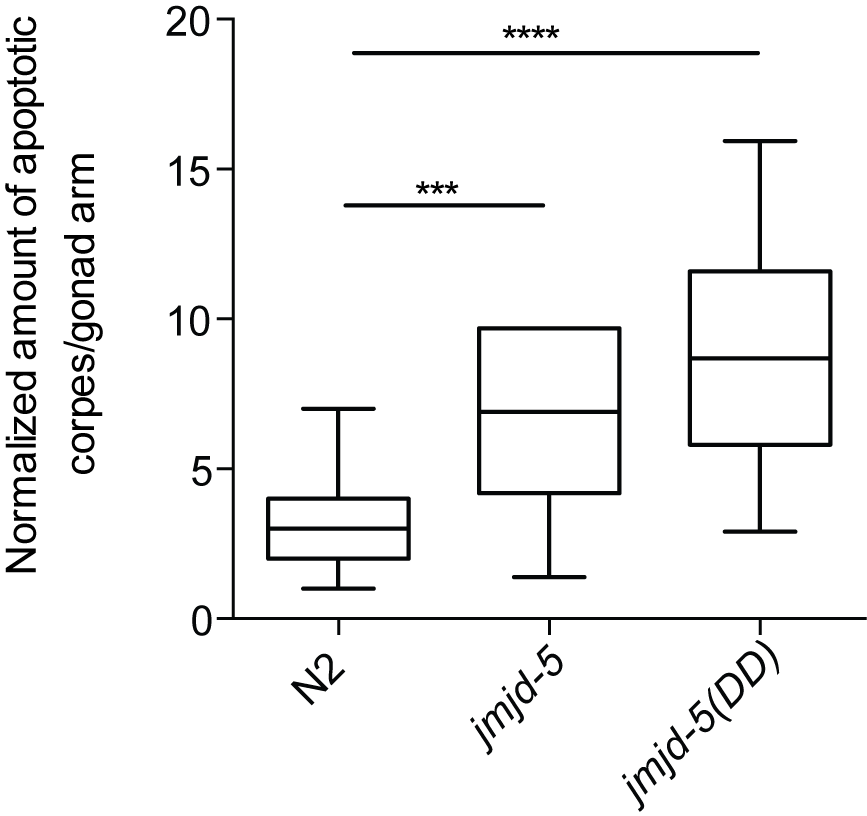

Supplement: S4 Fig — Quantification of apoptotic germ cells in the indicated strains grown at 25°C for five generations, using SYTO-12 staining. The average amount of SYTO12-labeled apoptotic corpses per gonad arm is normalized to the average number of meiotic germ cells in each of the indicated genotypes. At least 20 animals were scored for each genotype. ****p< 0.0001, ***p< 0.005 with two tailed unpaired t-test. (TIF) [file pgen.1006632.s004.tif]

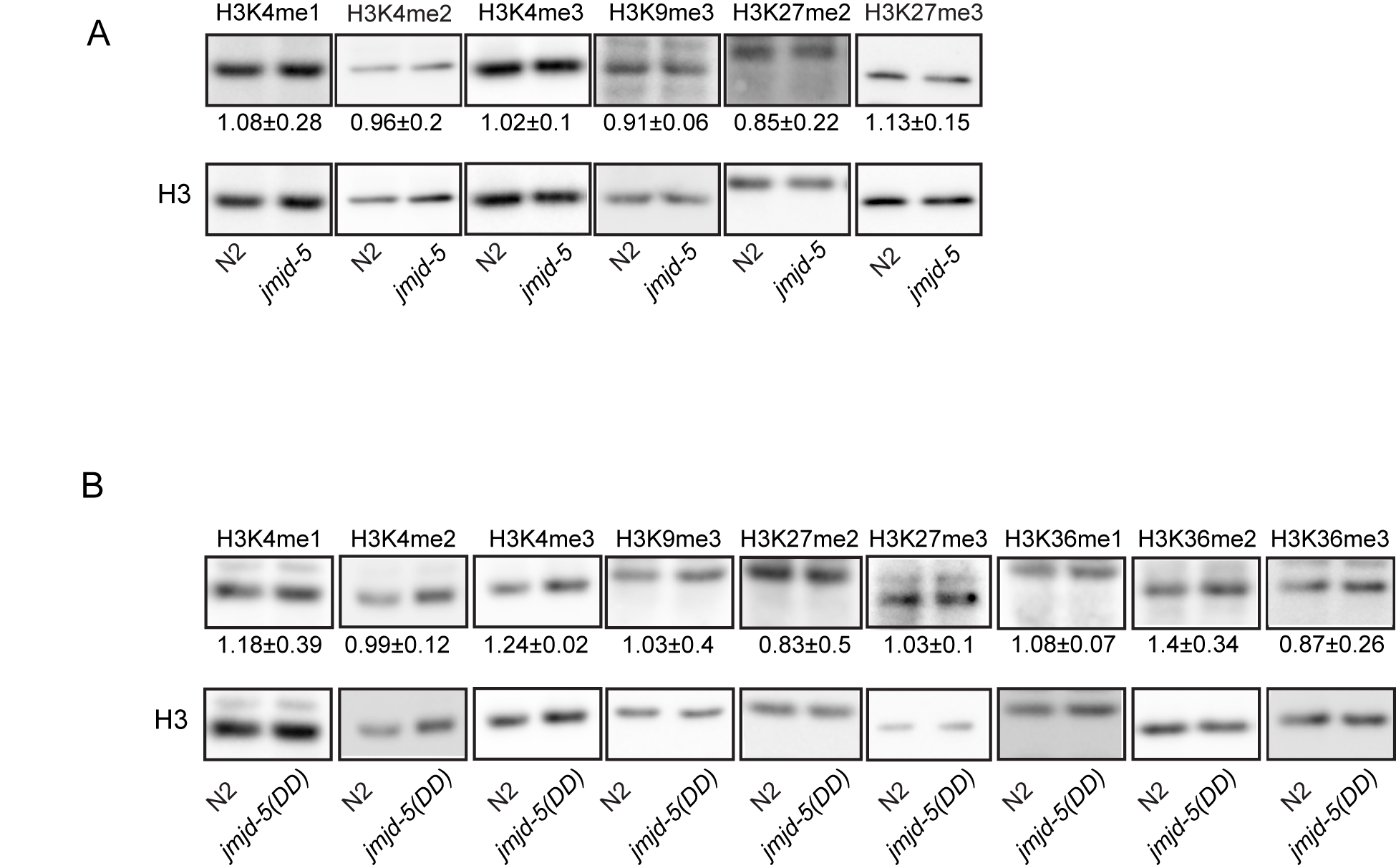

Supplement: S5 Fig — (A) Representative Western blots of the indicated histone modifications using lysates from N2 and jmjd-5(tm3735) animals. (B) Western blots of the indicated histone modifications using lysates from N2 and jmjd-5(DD) animals. H3 is used as loading control. Quantified levels of the histone modifications in jmjd-5 mutants relative to N2, are indicated. Quantification was performed using ImageJ. ± indicates SD, calculated from at least two independent experiments. (TIF) [file pgen.1006632.s005.tif]

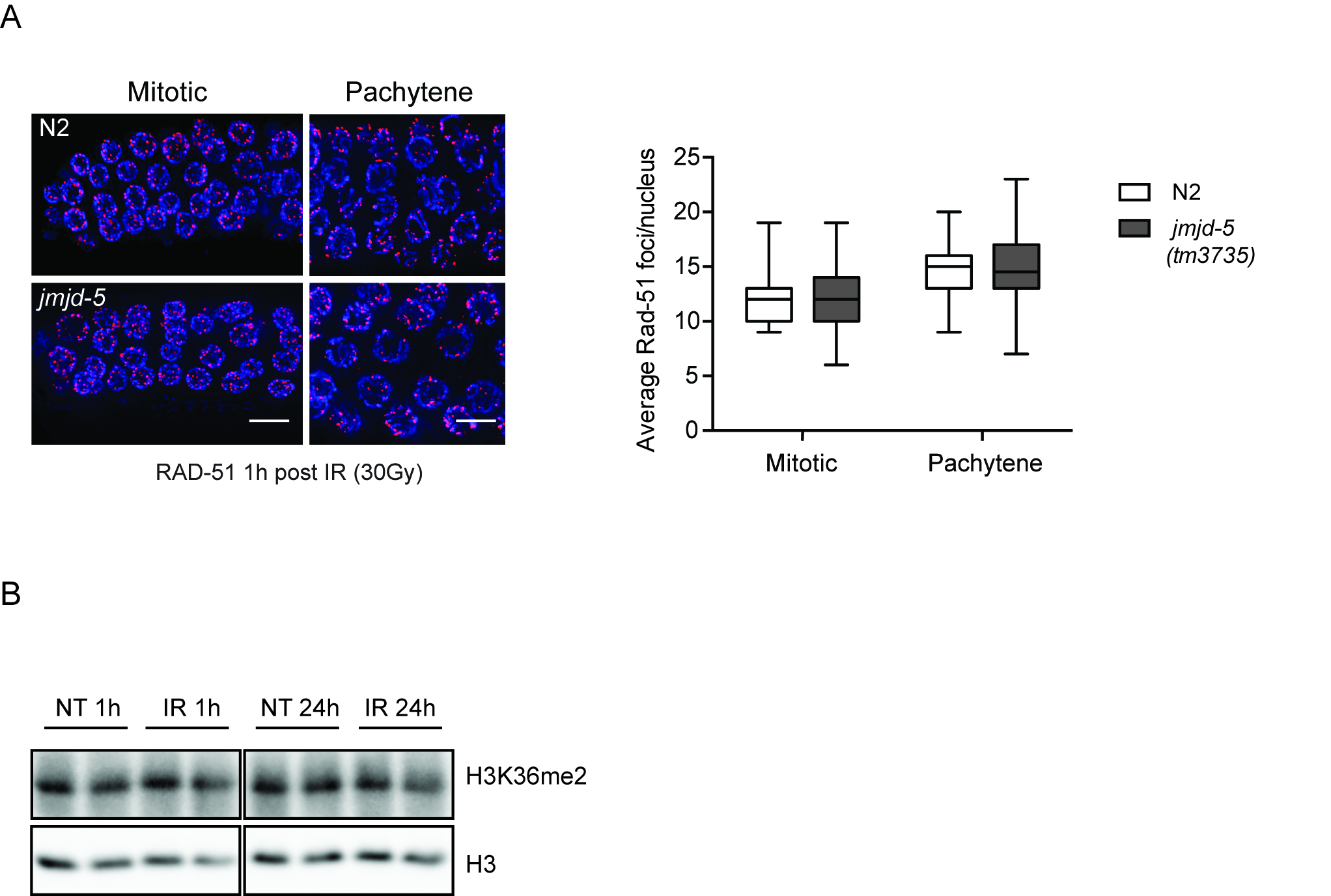

Supplement: S6 Fig — (A) Left: Representative images of RAD-51 (red) and DAPI (blue) staining in dissected germlines of N2 and jmjd-5(tm3735) 1 hour after IR (30 Gy). Mitotic and pachytene regions are shown. Each panel shows a projection of multiple z-stacks (0.2 μm spacing) of the entire nuclei. 100X magnification, scale bar 5 μm. Right: Quantification of RAD-51 foci per nucleus in N2 and jmjd-5(tm3735) 1 hour after IR (30Gy) in mitotic and pachytene regions. At least 4 germlines were analyzed. (B) Western blots showing the global level of H3K36me2 in N2 treated (IR) or not (NT) with 80Gy. Animals were collected after 1 and 24 hours of the treatment. H3 is used as loading control. Samples from two independent experiments are presented. (TIF) [file pgen.1006632.s006.tif]

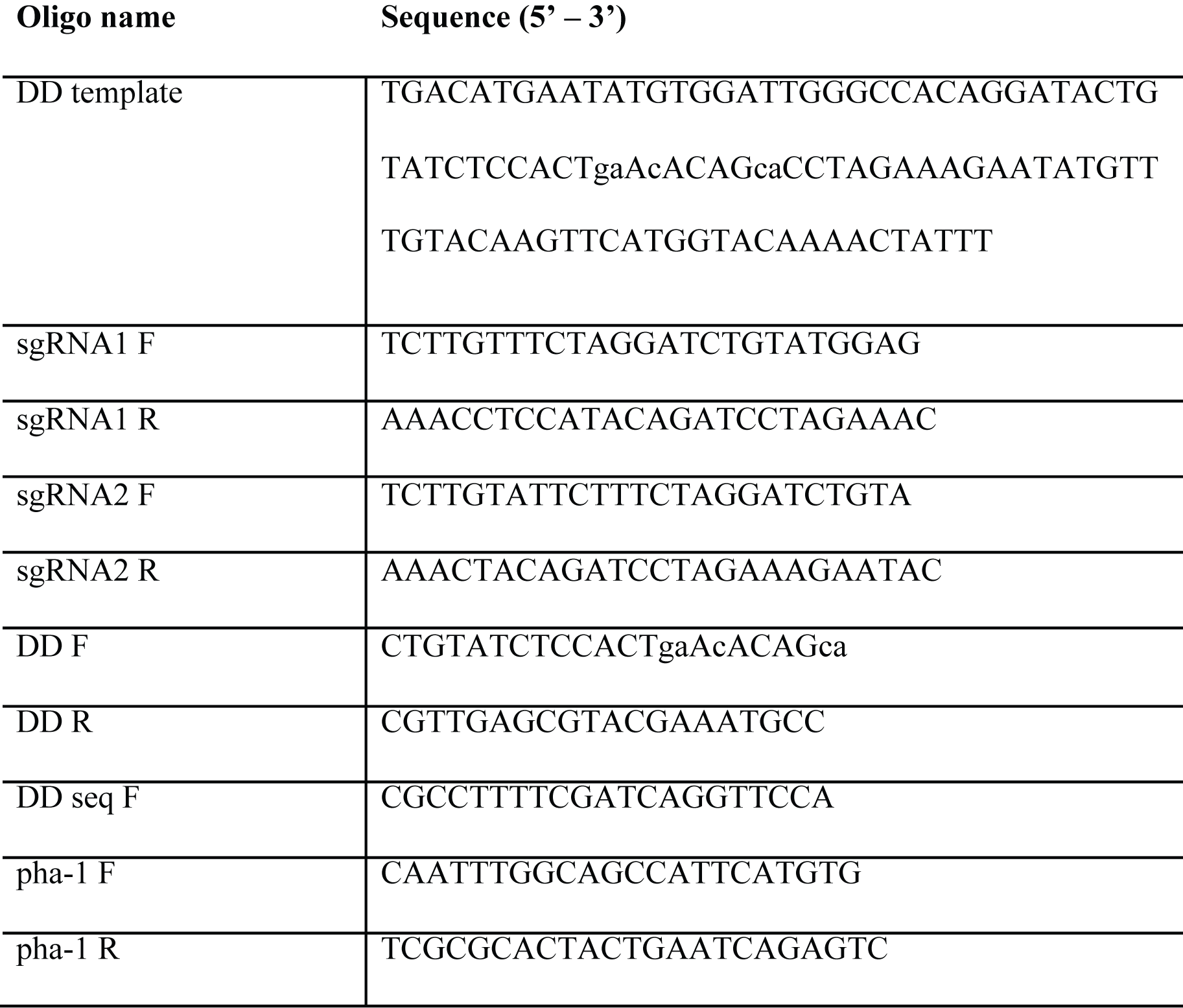

Supplement: S1 Table — (TIF) [file pgen.1006632.s007.tif]
